# Low Levels of Amyloid Precursor Protein (APP) Promote Neurogenesis and Decrease Gliogenesis in Human Neural Stem Cells

**DOI:** 10.3390/ijms241914635

**Published:** 2023-09-27

**Authors:** Raquel Coronel, Victoria López-Alonso, Marta I. Gallego, Isabel Liste

**Affiliations:** 1 Unidad de Regeneración Neural, Unidad Funcional de Investigación de Enfermedades Crónicas, Instituto de Salud Carlos III, 28220 Majadahonda, Madrid, Spain; 2 Departamento de Biología de Sistemas, Facultad de Medicina y Ciencias de la Salud, Universidad de Alcalá, 28871 Alcalá de Henares, Madrid, Spain; 3 Unidad de Biología Computacional, Unidad Funcional de Investigación de Enfermedades Crónicas, Instituto de Salud Carlos III, 28220 Majadahonda, Madrid, Spain; victorialopez@isciii.es; 4 Unidad de Histología y Patología Mamaria, Instituto de Salud Carlos III, 28220 Majadahonda, Madrid, Spain; migallego@isciii.es

**Keywords:** amyloid precursor protein, human neural stem cells, neurogenesis, gliogenesis, cell fate specification, proliferation

## Abstract

Amyloid precursor protein (APP) has been widely studied due to its association with Alzheimer’s disease (AD). However, the physiological functions of APP are still largely unexplored. APP is a transmembrane glycoprotein whose expression in humans is abundant in the central nervous system. Specifically, several studies have revealed the high expression of APP during brain development. Previous studies in our laboratory revealed that a transient increase in APP expression induces early cell cycle exit of human neural stem cells (hNSCs) and directs their differentiation towards glial cells (gliogenesis) while decreasing their differentiation towards neurons (neurogenesis). In the present study, we have evaluated the intrinsic cellular effects of APP down-expression (using siRNA) on cell death, cell proliferation, and cell fate specification of hNSCs. Our data indicate that APP silencing causes cellular effects opposite to those obtained in previous APP overexpression assays, inducing cell proliferation in hNS1 cells (a model line of hNSCs) and favoring neurogenesis instead of gliogenesis in these cells. In addition, we have analyzed the gene and protein expression levels of β-Catenin as a possible molecule involved in these cellular effects. These data could help to understand the biological role of APP, which is necessary to deepen the knowledge of AD.

## 1. Introduction

Alzheimer’s disease (AD) is the most common cause of dementia in the elderly population, affecting more than 44 million people worldwide. This disease currently has no cure and has been recognized by the World Health Organization (WHO) as a global public health problem [1].

Over the last 20 years, the amyloid cascade hypothesis has been the focus of research in relation to AD. This hypothesis postulates that the neurotoxic and neurodegenerative process observed in this disease is caused by the accumulation and aggregation of amyloid-β (Aβ) peptide in the brain parenchyma, forming amyloid plaques [2]. Aβ peptide is one of the main proteolytic derivatives of amyloid precursor protein (APP) that arises from proteolytic cleavage of this protein by β- and γ- secretases [3]. In recent years, several authors have defended that the absolute approach in the study of Aβ peptide (as a cause of amyloid plaques and AD) has resulted in APP itself, as well as its other proteolytic derivatives, being obviated in terms of possible therapeutic strategies of the disease [4].

More and more studies defend that APP contributes to AD’s pathological progression. Most of the mutations described in the *APP* gene are associated with familial AD (also known as EOAD), which represents 5% of cases and is characterized by having an early onset (symptoms appear in patients before age 65) [5]. In addition, several studies have shown that APP causes, at a cellular level, the deregulation of several signaling pathways, generating cellular and molecular alterations typical of AD [6]. However, the molecular mechanisms involved in the onset and during the course of AD are still not fully understood, and this is mainly because the physiological functions of APP are still poorly understood [7].

APP is an evolutionarily highly conserved transmembrane glycoprotein whose expression in humans is ubiquitous and abundant in the central nervous system [7,8]. In the embryo, APP expression is elevated during the early stages of nervous system development [9,10], suggesting that this protein plays an important role in neuronal growth and maturation [11]. Likewise, APP is abundantly expressed in the adult brain, and there are several functions associated with it, including its role in axonal growth and synaptic transmission, its involvement in cell differentiation and proliferation, its role in cell survival and neuronal maturation, as well as its role in cell adhesion [12,13,14].

In our previous study, we aimed to identify the intrinsic effects of APP in the differentiation of human neural stem cells (hNSCs). Our results have shown that a transient increase in the expression of APP induced early cell cycle exit of these cells and instructively directed their differentiation towards glial cells (gliogenesis), simultaneously preventing the differentiation of these cells towards neurons (neurogenesis) [15]. 

With the aim of providing consistency to previous data and in order to deepen the knowledge of the physiological function of APP, in the present study, we have evaluated the intrinsic cellular effects of APP down-expression (using small interfering RNA, siRNA) on cell death, cell proliferation, cell population of neural precursors, and cell fate specification of hNSCs. In addition, we have analyzed the gene and protein expression levels of β-Catenin as a possible molecule involved in the cellular effects previously observed.

## 2. Results

In this study, we used hNS1 cells as a model of hNSCs. This cell line has been previously characterized [15,16,17].

**Figure 1:**
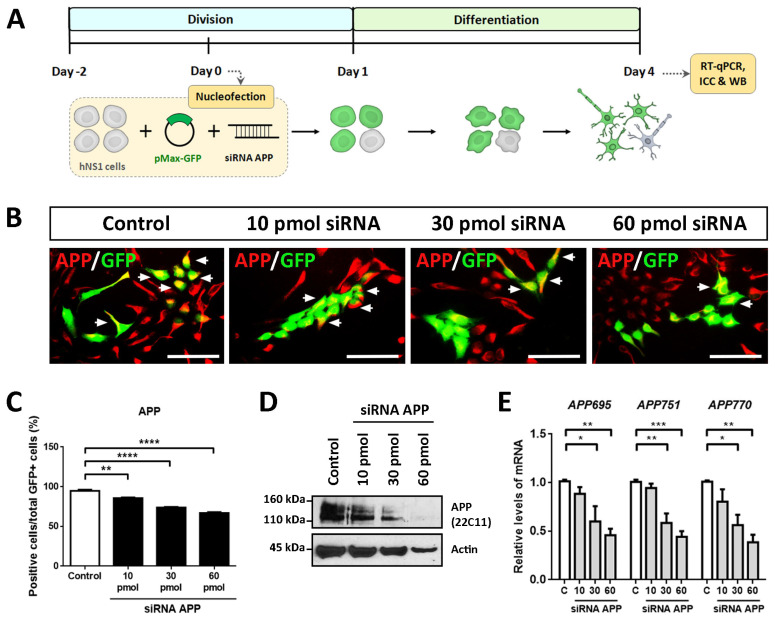
APP down-expression in hNS1 cells after transient nucleofection. (**A**) Schematic representation of the co-nucleofection experiment (pMax-GFP and siRNA APP) performed on hNS1 cells and the protocol used during cell proliferation (division) and differentiation (day 4). (**B**) Representative images at day 4 of differentiation by immunocytochemistry (ICC), showing immunoreactivity for APP in control hNS1 cells and hNS1 cells with APP silencing (10 pmol, 30 pmol, 60 pmol siRNA). White arrows indicate examples of co-localization between APP and GFP. Scale bar = 50 μm. (**C**) Percentage of GFP+ cells stained for APP relative to the total GFP+ cells in all study groups (control, 10 pmol, 30 pmol, and 60 pmol siRNA) on day 4 of differentiation. (**D**) Western blot (WB) assay against APP in cellular extracts of control hNS1 cells and hNS1 cells with APP silencing (10 pmol, 30 pmol, 60 pmol siRNA) at day 4 of differentiation. β-Actin was used as a loading control. (**E**) Relative expression levels of *APP695*, *APP751*, and *APP770* mRNA using quantitative real-time PCR (RT-qPCR) in all study groups (control, 10 pmol, 30 pmol, and 60 pmol siRNA) on day 4 of differentiation. *TBP* was used as a housekeeping gene. Data represent the mean ± SD (*n* = 3 for three independent samples). Statistical analysis was performed using one-way ANOVA (multiple comparisons of each group vs. the control group). * *p* < 0.05; ** *p* < 0.01; *** *p* < 0.001; **** *p* < 0.0001.

To evaluate the intrinsic effects of APP in hNS1 cells, we carried out loss-of-function studies by transient silencing of APP (using siRNA against human APP). For this, proliferating hNS1 cells were co-nucleofected with different amounts of siRNA (10 pmol, 30 pmol, and 60 pmol) targeted against APP and plasmid coding for GFP (to track transfected cells). After transfection, hNS1 cells of all study groups (control, 10 pmol, 30 pmol, and 60 pmol siRNA) were differentiated until day 4 (Figure 1A). We decided to perform these studies on a short time scale to avoid the appearance of possible compensatory effects that could result from stable APP down-expression. In addition, the previous studies were also evaluated on day 4 of differentiation, which allows us to compare them.

Nucleofection efficiency determined on day 1 post-nucleofection in all study groups was equivalent (between 35 and 40% of GFP+ cells) (Supplementary Figure S1). Subsequently, we performed ICC, RT-qPCR, and WB assays on day 4 of differentiation with the purpose of confirming the silencing of APP in the hNS1 cells.

The results obtained by ICC showed a gradual decrease in the immunoreactivity for APP according to the amount of siRNA APP assayed, being the weakest immunolabeling in the 60 pmol siRNA group compared to the control group (Figure 1B). These results were quantified by tracking the GFP+ cells, observing a significant decrease in the APP+/GFP+ cells in 10 pmol siRNA (85.6 ± 1.4%), 30 pmol siRNA (73.7 ± 1.3%), and 60 pmol siRNA (66.8 ± 2.0%) compared to the control group (94.6 ± 3.0%) (Figure 1C). Likewise, these data were confirmed at the protein level by WB analysis (Figure 1D) and at the mRNA level (assessing gene expression of *APP695*, *APP751*, and *APP770*, majority isoforms of APP) by RT-qPCR (Figure 1E).

After confirming the transient silencing of APP in the hNS1 cells, we evaluated the effects of APP down-expression on several cellular biological processes.

### 2.1. APP Down-Expression Effects on Cell Death

**Figure 2:**
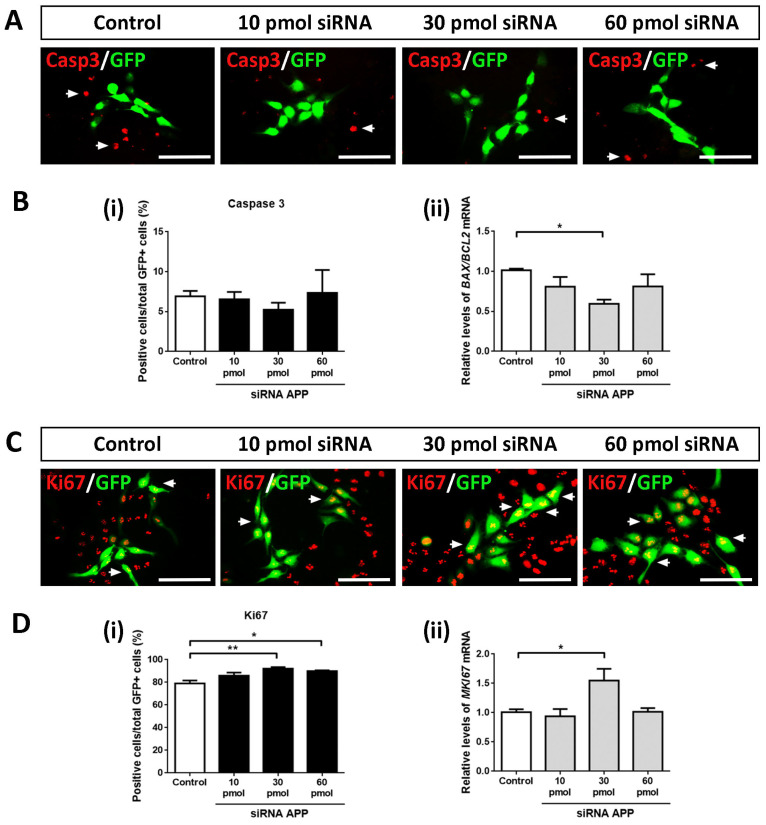
Effects of APP down-expression on cell death and cell proliferation of the hNS1 cells. (**A**) Representative images at day 4 of differentiation by ICC, showing immunoreactivity for Caspase 3 (activated) in control hNS1 cells and hNS1 cells with APP silencing (10 pmol, 30 pmol, and 60 pmol siRNA). Scale bar = 50 μm. (**B**) (**i**) Percentage of GFP+ cells stained for Caspase 3 relative to the total GFP+ cells in all study groups (control, 10 pmol, 30 pmol, and 60 pmol siRNA) on day 4 of differentiation. (**B**) (**ii**) Relative expression levels of *BAX*/*BCL2* mRNA using RT-qPCR in control hNS1 cells and hNS1 cells with APP silencing (10 pmol, 30 pmol, and 60 pmol siRNA) at day 4 of differentiation. *TBP* was used as a housekeeping gene. (**C**) Representative images at day 4 of differentiation by ICC, showing immunoreactivity for Ki67 in all study groups (control, 10 pmol, 30 pmol, and 60 pmol siRNA) on day 4 of differentiation. White arrows indicate examples of co-localization between APP and GFP. Scale bar = 50 μm. (**D**) (**i**) Percentage of GFP+ cells stained for Ki67 relative to the total GFP+ cells in control hNS1 cells and hNS1 cells with APP silencing (10 pmol, 30 pmol, and 60 pmol siRNA) at day 4 of differentiation. (**D**) (**ii**) Relative expression levels of *MKI67* mRNA by RT-qPCR in all study groups (control, 10 pmol, 30 pmol, and 60 pmol siRNA) on day 4 of differentiation. *TBP* was used as a housekeeping gene. Data represent the mean ± SD (*n* = 3 for three independent samples). Statistical analysis was performed using one-way ANOVA (multiple comparisons of each group vs. the control group). * *p* < 0.05; ** *p* < 0.01.

First, we analyzed whether APP silencing (at the amounts of siRNA tested) affected the apoptotic cell death of hNS1 cells. For this, we performed ICC assays against activated Caspase 3 (a marker of cell death by apoptosis) on day 4 of differentiation in all study groups (control, 10 pmol, 30 pmol, and 60 pmol siRNA). After tracking the GFP+ cells, we did not observe statistically significant differences in the Caspase 3+/GFP+ cells between all study groups, indicating that, at the amounts of siRNA tested, APP silencing does not affect apoptotic cell death in the hNS1 cells (Figure 2A,B(i)). Similarly, we analyzed the gene expression levels of *BAX* (pro-apoptotic gene) and *BCL2* (anti-apoptotic gene), and we performed the *BAX*/*BCL2* ratio (an indicator of cell apoptosis) at the mRNA level without obtaining statistically significant differences between the studied groups (except in the comparison of the 30 pmol siRNA group versus the control group) (Figure 2B(ii)). 

Therefore, we conclude that, at the level of transfected cells, APP silencing does not affect apoptotic cell death in hNS1 cells.

### 2.2. APP Down-Expression Effects on Cell Proliferation 

Subsequently, we evaluated the possible intrinsic effects of APP silencing on the cell cycle. To do this, we performed ICC assays against Ki67 (a cell cycle marker) on day 4 of differentiation in control hNS1 cells and hNS1 cells with APP silencing (10 pmol, 30 pmol, and 60 pmol siRNA) (Figure 2C). After counting the GFP+ cells, we observed a significant increase in Ki67+/GFP+ cells at the 30 pmol (92.2 ± 2.0%) and 60 pmol groups (89.9 ± 1.5%) compared to the control group (79.1 ± 4.5%) (Figure 2D(i)). These results were confirmed at the mRNA level by analyzing the gene expression of *MKI67* using RT-qPCR, obtaining statistically significant differences in the comparison of the 30 pmol siRNA group versus the control group (Figure 2D(ii)).

These data indicate that, at the intrinsic level, APP silencing increases the number of cells in the cell cycle state, which is equivalent to an increase in the proliferation of hNS1 cells.

### 2.3. APP Down-Expression Effects on Cell Population of Neural Precursors

In the same way, we analyzed the possible intrinsic effects of APP silencing on the cell population of neural precursors to determine if these were affected by the increase in cell proliferation (previously observed). To do this, we again evaluated using ICC assays against Nestin and Sox2 (both markers of neural precursors) on day 4 of differentiation in all study groups (control, 10 pmol, 30 pmol, and 60 pmol siRNA). 

**Figure 3:**
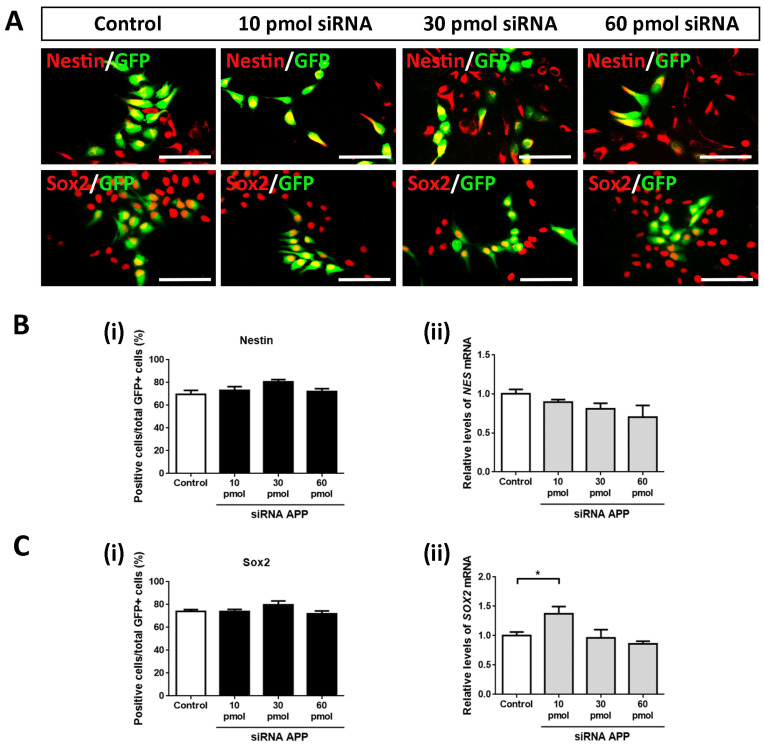
Effects of APP down-expression on the cell population of neural precursors of hNS1 cells. (**A**) Representative images at day 4 of differentiation by ICC, showing immunoreactivity for Nestin (upper panels) and Sox2 (lower panels) in control hNS1 cells and hNS1 cells with APP silencing (10 pmol, 30 pmol, and 60 pmol siRNA). Scale bar = 50 μm. (**B**) (**i**) Percentage of GFP+ cells stained for Nestin relative to the total GFP+ cells in all study groups (control, 10 pmol, 30 pmol, and 60 pmol siRNA) on day 4 of differentiation. (**B**) (**ii**) Relative expression levels of *NES* mRNA by RT-qPCR in control hNS1 cells and hNS1 cells with APP silencing (10 pmol, 30 pmol, and 60 pmol siRNA) at day 4 of differentiation. *TBP* was used as a housekeeping gene. (**C**) (**i**) Percentage of GFP+ cells stained for Sox2 relative to the total GFP+ cells in all study groups (control, 10 pmol, 30 pmol, and 60 pmol siRNA) on day 4 of differentiation. (**C**) (**ii**) Relative expression levels of *SOX2* mRNA by RT-qPCR in the control hNS1 cells and hNS1 cells with APP silencing (10 pmol, 30 pmol, and 60 pmol siRNA) at day 4 of differentiation. *TBP* was used as a housekeeping gene. Data represent the mean ± SD (*n* = 3 for three independent samples). Statistical analysis was performed using one-way ANOVA (multiple comparisons of each group vs. the control group). * *p* < 0.05.

Contrary to expectations, in this case, we did not obtain statistically significant differences in Nestin+/GFP+ cells (Figure 3A,B(i)) nor in Sox2+/GFP+ cells (Figure 3A,C(i)), maintaining the cell population of neural precursors in a high and constant proportion (between 70 and 80% of Nestin+/GFP+ cells and Sox2+/GFP+ cells) in all study groups. However, it is important to highlight the slightly increasing trend in both cases. These results were confirmed at the mRNA level by analyzing the gene expression of *NES* and *SOX2* by RT-qPCR (except for the comparison of the 10 pmol siRNA group versus the control group) (Figure 3B(ii),C(ii)).

Together with these data, we conclude that, at the level of transfected cells, APP silencing increases cell proliferation in hNS1 cells without apparently affecting the cell population of the neural precursor (probably due to their early cell differentiation).

### 2.4. APP Down-Expression Effects on Cell Fate Specification

After analyzing the intrinsic effects of APP silencing on apoptotic cell death, cell proliferation, and cell population of neural precursors, we studied whether APP down-expression affected the phenotypic specification of hNS1 cells (as we observed in previous gain-of-function studies by the transient overexpression of APP) [15].

**Figure 4:**
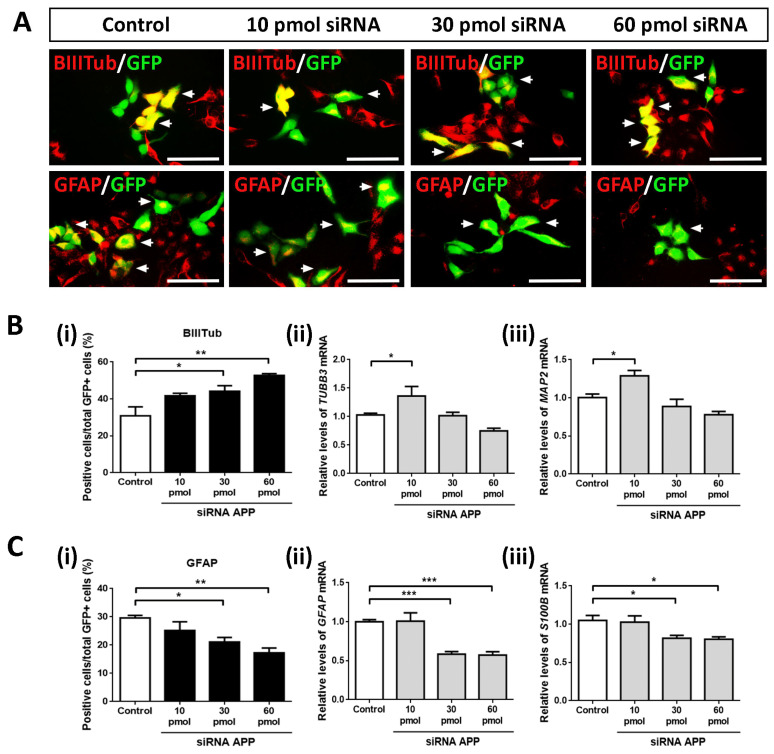
Effects of APP down-expression on the cell fate specification of hNS1 cells. (**A**) Representative images at day 4 of differentiation by ICC, showing immunoreactivity for β-III-tubulin (BIIITub; upper panels) and GFAP (lower panels) in control hNS1 cells and hNS1 cells with APP silencing (10 pmol, 30 pmol, and 60 pmol siRNA). White arrows indicate examples of co-localization between APP and GFP. Scale bar = 50 μm. (**B**) (**i**) Percentage of GFP+ cells stained for β-III-tubulin relative to the total GFP+ cells in all study groups (control, 10 pmol, 30 pmol, and 60 pmol siRNA) on day 4 of differentiation. (**B**) (**ii**,**iii**) Relative expression levels of *TUBB3* (**ii**) and *MAP2* (**iii**) mRNA using RT-qPCR in control hNS1 cells and hNS1 cells with APP silencing (10 pmol, 30 pmol, and 60 pmol siRNA) at day 4 of differentiation. *TBP* was used as a housekeeping gene. (**C**) (**i**) Percentage of GFP+ cells stained for GFAP relative to the total GFP+ cells in all study groups (control, 10 pmol, 30 pmol, and 60 pmol siRNA) on day 4 of differentiation. (**C**) (**ii**,**iii**) Relative expression levels of *GFAP* (**ii**) and *S100B* (**iii**) mRNA using RT-qPCR in control hNS1 cells and hNS1 cells with APP silencing (10 pmol, 30 pmol, and 60 pmol siRNA) at day 4 of differentiation. *TBP* was used as a housekeeping gene. Data represent the mean ± SD (*n* = 3 for three independent samples). Statistical analysis was performed using one-way ANOVA (multiple comparisons of each group vs. the control group). * *p* < 0.05; ** *p* < 0.01; *** *p* < 0.001.

To this end, we performed ICC assays against β-III-tubulin (neuronal marker) and GFAP (glial cell marker) on day 4 of differentiation in control hNS1 cells and hNS1 cells with APP silencing (10 pmol, 30 pmol, and 60 pmol siRNA) (Figure 4A). After tracking the GFP+ cells, we observed a gradual and significant increase (according to the amount of siRNA APP assayed) in β-Tubulin-III+/GFP+ cells at the 30 pmol (44.2 ± 5.3%) and 60 pmol groups (52.7 ± 1.7%) compared to the control group (30.8 ± 8.5%) (Figure 4B(i)). These results were confirmed at the mRNA level by analyzing the gene expression of *TUBB3* and *MAP2* (gene encoding MAP2; neuronal marker) using RT-qPCR, obtaining statistically significant differences in the comparison of the 10 pmol siRNA group versus the control group (Figure 4B(ii,iii)).

On the contrary, we detected a significant and progressive decrease (according to the amount of siRNA APP assayed) in the GFAP+/GFP+ cells at the 30 pmol (21.1 ± 2.8%) and 60 pmol groups (17.2 ± 3.0%) compared to the control group (29.6 ± 1.6%) (Figure 4C(i)). Likewise, these results were confirmed at the mRNA level by analyzing the gene expression of *GFAP* and *S100B* (gene encoding S100β; astrocyte marker) using RT-qPCR (Figure 4C(ii,iii)).

All these results indicate that, at an intrinsic level, the silencing of APP favors the differentiation of hNS1 cells towards a neuronal phenotype (neurogenesis); at the same time, it decreases their differentiation towards glial cells (gliogenesis). Both effects are opposite to those we obtained in previous APP gain-of-function studies (increased gliogenesis and decreased neurogenesis), confirming our conclusions about APP’s dual role in cell fate specification.

### 2.5. Molecular Effects of APP Down-Expression

Due to the previously described effects of APP down-expression on the biology of hNS1 cells and the consulted publications (regarding molecules involved in cell differentiation of neural stem cells (NSCs)) [18,19,20], we decided to evaluate the presence of β-Catenin in our experiments of APP loss-of-function. For this, we conducted ICC, RT-qPCR, and WB assays in hNS1 cells with APP silencing at day 4 of differentiation.

**Figure 5:**
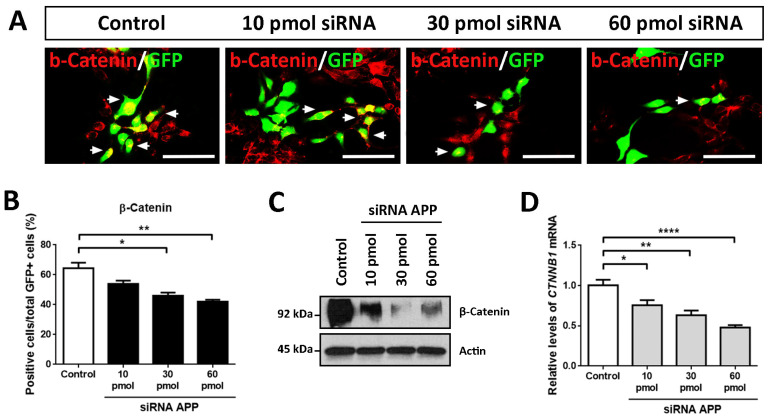
Molecular effects of APP down-expression in hNS1 cells. (**A**) Representative images at day 4 of differentiation by ICC, showing the immunoreactivity for β-Catenin in control hNS1 cells and hNS1 cells with APP silencing (10 pmol, 30 pmol, and 60 pmol siRNA). White arrows indicate examples of co-localization between APP and GFP. Scale bar = 50 μm. (**B**) Percentage of GFP+ cells stained for β-Catenin relative to the total GFP+ cells in all study groups (control, 10 pmol, 30 pmol, and 60 pmol siRNA) on day 4 of differentiation. (**C**) WB assay against β-Catenin in cellular extracts of control hNS1 cells and hNS1 cells with APP silencing (10 pmol, 30 pmol, and 60 pmol siRNA) at day 4 of differentiation. β-Actin was used as a loading control. (**D**) Relative expression levels of *CTNNB1* mRNA by RT-qPCR in all study groups (control, 10 pmol, 30 pmol, 60 pmol siRNA) on day 4 of differentiation. *TBP* was used as a housekeeping gene. Data represent the mean ± SD (*n* = 3 for three independent samples). Statistical analysis was performed using one-way ANOVA (multiple comparisons of each group vs. the control group). * *p* < 0.05; ** *p* < 0.01; **** *p* < 0.0001.

The results obtained by ICC showed a gradual decrease in the immunoreactivity for β-Catenin according to the amount of siRNA APP assayed, being the weakest immunolabeling in the 60 pmol siRNA group compared to the control group (Figure 5A). These results were quantified by tracking the GFP+ cells, observing a significant decrease in β-Catenin+/GFP+ cells at the 30 pmol (46.0 ± 3.0%) and 60 pmol groups (42.0 ± 1.9%) compared to the control group (64.4 ± 5.3%) (Figure 5B).

In addition, these data were confirmed at the protein level by WB analysis (Figure 5C) and at the mRNA level (*CTNNB1* is the gene encoding for β-Catenin) by RT-qPCR (Figure 5D). In these analyses, statistically significant differences were obtained in all the comparisons of the study groups with APP silencing (10 pmol, 30 pmol, and 60 pmol siRNA) compared to the control group.

Altogether from these data, we conclude that, at the molecular level, APP silencing affects the protein levels of β-Catenin, as well as its mRNA levels (*CTNNB1*), which both decrease according to the amount of siRNA APP tested in hNS1 cells. For all these reasons, we think that β-Catenin could be involved, in one way or another, in the previously described cellular effects.

## 3. Discussion

**Figure 6:**
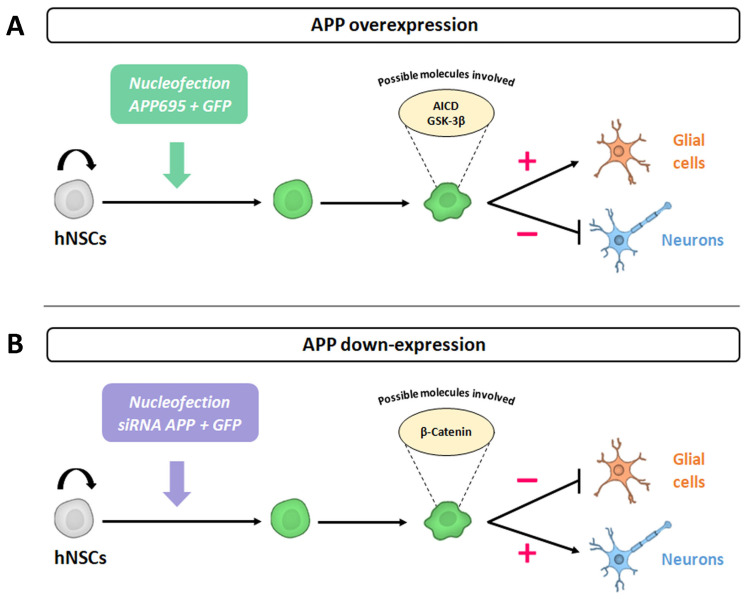
Scheme illustrating the possible effects of APP on cell fate specification of hNSCs. (**A**) Previous studies in our laboratory revealed that APP overexpression favors the gliogenesis process and prevents the neurogenesis in hNS1 cells. We proposed to APP intracellular C-terminal domain (AICD) and GSK-3β as possible molecules involved in the observed effects. (**B**) On the contrary, current studies in our laboratory show that APP down-expression favors neurogenesis instead of gliogenesis in hNS1 cells. In this case, we suggest to β-Catenin as a possible molecule involved in the observed effects.

In previous studies, we observed that a transient increase in the expression of APP at an intrinsic level favored the gliogenesis process, and simultaneously, it prevented the neurogenesis process in hNSCs (hNS1 cell line) [15]. On the contrary, in this work, we have observed that a decrease in the expression of APP, also at the intrinsic level, favors the differentiation of hNS1 cells towards a neuronal phenotype (neurogenesis) and prevents their glial differentiation (gliogenesis) (Figure 6).

Several authors have proposed that APP and its proteolytic derivatives are capable of regulating the phenotypic specification of hNSCs [21,22]. It has been described that in vitro differentiation of primary cultures of NSCs (isolated from *App-KO* mice) causes an increase in the generation of new neurons (neurogenesis) [23]. In addition, the transfection of siRNA APP into murine NSCs increases cell proliferation [24], consistent with the results obtained in our APP silencing assays in hNS1 cells.

Likewise, APP loss-of-function assays in hNSCs (generated from human induced pluripotent stem cells (hiPSCs) *APP-KO* using CRISPR/Cas9) show an increase in neuronal differentiation and even an acceleration in the appearance of the cortical phenotype [25], which reaffirms our previous conclusions regarding the neurogenesis process. In relation to these data, the elimination of APP expression should not be taken as a potential therapy for certain neurodegenerative diseases (such as AD). It has also been described that the loss of APP expression (in *App-KO* mice) presents aberrant effects on hippocampal neuronal development (such as morphological abnormalities in dendritic and axonal growth), leading to synaptogenic alterations [26].

Hence, we believe that the maintenance of APP at adequate expression levels is essential for proper cell function and, particularly, to play its role in the cell fate specification of hNSCs.

On the other hand, in relation to the possible molecular mechanisms involved in the cellular effects previously described by APP down-expression, our results clearly indicate that APP silencing in hNS1 cells gradually decreases the gene and protein expression of β-Catenin (according to the amount of siRNA against APP tested).

β-Catenin is a key factor in the canonical Wnt signaling pathway. In the absence of the Wnt ligand (inactive pathway), β-Catenin is phosphorylated by a protein complex (formed by Axin, APC, and GSK-3β) and degraded by proteasome [27,28]. On the contrary, in the presence of the Wnt ligand (active pathway), β-Catenin is not phosphorylated and is translocated to the cell nucleus, where it acts by regulating the transcription of a wide variety of target genes [27,28].

In vitro studies of primary rat neurons with stable APP overexpression showed a decrease in total β-Catenin protein levels and an increase in phosphorylated β-Catenin levels, indicating that APP facilitates the proteasomal degradation of β-Catenin. Similarly, the same authors detected opposite effects (increase in total β-Catenin and decrease in phosphorylated β-Catenin) in rat primary neurons with stable APP silencing (by short hairpin RNA, shRNA) [29]. In addition, the authors also observed that APP overexpression drastically decreased the presence of β-Catenin at membrane and cytoplasmic levels without affecting β-Catenin located in the cell nucleus, suggesting a differential function of this protein (according to their subcellular location) in response to APP.

Although these results could (apparently) be contradictory to those obtained in our APP loss-of-function assays in hNS1 cells (where transient APP silencing causes a decrease in total β-Catenin levels), we think that these effects could be due to differences in the study model (rat primary neurons versus hNSCs) and the type of silencing (stable versus transient).

It has also been described that APP not only influences β-Catenin levels but is also capable of forming a protein complex directly with this protein (APP-β-Catenin) and modulating its subcellular localization. In this way, it has been detailed that transient overexpression of APP in N2a cells (murine neuroblastoma cell line) prevents the translocation of β-Catenin to the cell nucleus and, therefore, the transcription of Wnt target genes [30].

Regarding whether the cellular effects observed (increased neurogenesis and decreased gliogenesis) in hNS1 cells with APP silencing could be mediated, directly or indirectly, by β-Catenin, there is evidence supporting a dynamic regulation model in the phenotypic specification of NSCs, controlled in turn by the dynamic expression of β-Catenin [18]. In these studies, the authors observed that a sustained signal from β-Catenin promotes neurogenesis, while transient signals from this protein induce apoptotic cell death. Furthermore, it has been described that the inhibition of the Wnt signaling pathway favors the differentiation of NSCs towards an astroglial phenotype, while the activation of the Wnt pathway (through the constitutive expression of β-Catenin) promotes neurogenesis [19].

According to our results, APP silencing promotes neurogenesis, at the same time that it decreases gliogenesis in hNS1 cells, in addition to decreasing the levels of gene and protein expression of β-Catenin. Thus, we think that changes in β-Catenin levels could be due to (1) β-Catenin being one of the molecules involved in regulating the cell fate specification of hNSCs and (2) β-Catenin being closely related to APP, with the decrease in β-Catenin levels being caused by the decrease in APP levels.

In conclusion, our data indicate a role of APP in controlling the cell proliferation and cell fate specification of hNSCs (increasing neurogenesis and decreasing gliogenesis), and these effects could be mediated by β-Catenin or be related to this protein. This finding may contribute to knowledge of APP physiological functions and elucidate the multiple roles of this protein, being essential and necessary to be able to advance our understanding of AD pathogenesis.

## 4. Materials and Methods

### 4.1. Ethics Statement

We used hNS1 cells, a model of hNSCs with the capacity to differentiate into neurons, astrocytes, and oligodendrocytes. These cells are derived from the forebrain of a 9.5 weeks gestational age human fetus, which were immortalized with *v-myc* by retroviral infection [16,17]. The hNS1 cell line has been previously characterized [15]. The original human fetal tissues were donated to the research after signing an informed consent. The tissues were obtained in accordance with the Declaration of Helsinki of the World Medical Assembly (WMA) and the ethical standards of the Network of European CNS Transplantation and Restoration (NECTAR). Approval to use these tissues for research was granted by the University of Lund Hospital Ethics Committee, and its use was carried out in accordance with Spanish Law 14/2007 on Biomedical Research. The study was approved by the Ethics Committee of the Instituto de Salud Carlos III (approval number PI93-2020).

### 4.2. Cell Culture and APP Down-Expression

hNS1 cells were cultured on plates pre-treated with poly-L-lysine (10 μg/mL, Sigma, St. Louis, MO, USA) and proliferated on a chemically defined human stem cell (HSC) medium supplemented with fibroblast growth factor (FGF2) (20 ng/mL, Peprotech, Waltham, MA, USA) and epidermal growth factor (EGF) (20 ng/mL, Peprotech). Proliferating hNS1 cells at 70–80% confluence were dissociated with trypsin (0.25%, Gibco, Waltham, MA, USA), centrifuged (4 min at 900 rpm), and nucleofected (Nucleofector Amaxa 2B, Lonza, Basel, Switzerland) according to the manufacturer’s instructions. Briefly, for the different groups with APP silencing, hNS1 cells were pre-mixed, in each case, with 10 pmol, 30 pmol, and 60 pmol of siRNA duplex against human APP (Santa Cruz Biotechnology, Dallas, TX, USA) in 100 µL of nucleofection solution (Lonza). Note that the concentration of siRNA APP used in each case corresponds to 100 nM, 300 nM, and 600 nM, respectively. Likewise, we previously tested a broader range of siRNA APP concentrations and detected a generalized cell death from 90 pmol (900 nM). Similarly, for the control group, hNS1 cells were pre-mixed with 60 pmol of control siRNA duplex (Santa Cruz Biotechnology) in 100 µL of nucleofection solution. In addition, in all study groups, hNS1 cells were co-transfected with 2 μg of pMax-GFP plasmid (Lonza) to track transfected cells. Then, hNS1 cells were plated in a proliferation medium, and after 1 day, they were differentiated on HSC medium (without growth factors) and heat-inactivated fetal bovine serum (FBS) (0.5%, Gibco) for 4 days. In all cases, the hNS1 cells were maintained in an incubator at 37 °C and 5% CO_2_ with relative humidity.

### 4.3. Immunocytochemistry

hNS1 cells cultivated in multiwell plates (at 4 days after nucleofection) were fixed with PFA (4%, Sigma) for 10 min. Subsequently, cultures were blocked with normal horse serum (NHS) (5%, Gibco) and triton X-100 (0.25%, Merck, Rahway, NJ, USA) in PBS for 30 min and incubated overnight at 4 °C with rabbit antibodies against Caspase 3 (activated; 1:500, Cell Signaling), Ki67 (1:500, Invitrogen, Waltham, MA, USA), Nestin (1:500, Sigma), Sox2 (1:500, Millipore, Burlington, MA, USA), β-Catenin (1:100, Cell Signaling, Danvers, MA, USA), or mouse antibodies against APP (22C11 clone; 1:500, Millipore), β-III-tubulin (class III β-tubulin; 1:3000, Biolegend, San Diego, CA, USA), and GFAP (1:1000, BD Pharmigen, Waltham, MA, USA). Then, cultures were washed with triton X-100 in PBS and incubated with Alexa 555-conjugated antibody (donkey anti-rabbit; 1:500, Invitrogen, or donkey anti-mouse; 1:500, Invitrogen) for 1 h in darkness. All antibodies were diluted in NHS (1%, Gibco) and triton X-100 in PBS. Visualization and analysis of preparations were performed using fluorescence microscopy (Leica DMIL LED, Wetzlar, Germany). Image analysis was performed using Photoshop CS6 after randomly capturing at least 10–12 separate fields per well (and at least 100 GFP+ cells were counted per well), with a minimum of three wells per study group (*n* = 3).

### 4.4. Western Blot

hNS1 cells cultivated in multiwell plates (at 4 days after nucleofection) were dissociated with trypsin and centrifuged in PBS. The pellets were treated with lysis buffer (RIPA, Cell Signaling) to obtain cell extracts. In all cases, 50 μg of total proteins from cell extracts were loaded on sodium dodecyl sulfate-polyacrylamide gels (10%, BioRad, Hercules, CA, USA), electrophoresed (SDS-PAGE), and transferred to nitrocellulose membranes (GE Healthcare). The membranes were blocked with milk (5%) and Tween20 (0.05%, Sigma) in TBS for 1 h and incubated overnight at 4 °C with rabbit antibodies against β-Catenin (1:1000, Cell Signaling) or mouse antibodies against APP (clone 22C11; 1:1000, Millipore) and β-Actin (1:1000, Sigma). Then, the membranes were washed with Tween20 in TBS and incubated with HRP-conjugated antibody (goat anti-rabbit peroxidase; 1:3000, Vector Laboratories, Newark, CA, USA, or horse anti-mouse peroxidase; 1:3000, Vector Laboratories) for 1 h. All the antibodies were diluted in milk and Tween20 in TBS. Visualization of immunoreactive bands was performed using an ECL chemiluminescent system (Millipore) according to the manufacturer’s instructions.

### 4.5. Quantitative Real-Time PCR

hNS1 cells cultivated in multiwell plates (at 4 days after nucleofection) were dissociated with trypsin and centrifuged in PBS. Total RNA from the pellets was isolated using a spin column kit (Rneasy Mini Kit, Qiagen, Germantown, MD, USA) according to the manufacturer’s instructions and treated with DNAses to avoid amplification of undesired genomic and plasmid DNA. Then, 1 µg of total RNA was reverse transcribed at 50 °C for 60 min using Superscript III reverse transcriptase (Invitrogen). Relative amounts of cDNA were quantified with quantitative real-time PCR using the SYBR Green fluorophore (PowerUp SYBR Green Master Mix, Applied Biosystems, Waltham, MA, USA) and QuantStudio 3 system (Applied Biosystems) according to the manufacturer’s protocol. In all cases, 10 ng of cDNA were amplified using primers for the follow human target genes: *APP770* (forward: 5′-TTGCCCGAGATCCTGTTAAA-3′; reverse: 5′-TACTTGTCAACGGCATCAGG-3′), *APP751* (forward: 5′-CGGAACAACTTTGACACAGAAG-3′; reverse: 5′-ACTTGTCAACGGCATCAGG-3′), *APP695* (forward: 5′-GACGATGAGGATGGTGATGA-3′; reverse: 5′-CTGGCTGCTGTTGTAGGAACT-3′), *BAX* (forward: 5′-AGCAAACTGGTGCTCAAGG-3′; reverse; 5′-CTTGGATCCAGCCCAACA-3′), *BCL2* (forward: 5′-TTGACAGAGGATCATGCTGTACT-3′; reverse; 5′-ATCTTTATTTCATGAGGCACGTT-3′), *MKI67* (forward: 5′-TGACCCTGATGAGAAAGCTCAA-3′; reverse; 5′-CCCTGAGCAACACTGTCTTTT-3′), *NES* (forward: 5′-GAGGTGGCCACGTACAGG-3′; reverse; 5′-AAGCTGAGGGAAGTCTTGGA-3′), *SOX2* (forward: 5′-GGGGGAATGGACCTTGTATAG-3′; reverse; 5′-GCAAAGCTCCTACCGTACCA-3′), *TUBB3* (forward: 5′-GCAACTACGTGGGCGACT-3′; reverse; 5′-ATGGCTCGAGGCACGTACT-3′), *MAP2* (forward: 5′-ATCTCTTCTTCAGCACGGCG-3′; reverse; 5′-CAGGGGTAGTGGGTGTTGAG-3′), *GFAP* (forward: 5′-GTTCTTGAGGAAGATCCACGA-3′; reverse; 5′-CTTGGCCACGTCAAGCTC-3′), *S100B* (forward: 5′-GGAAGGGGTGAGACAAGGA-3′; reverse; 5′-GGTGGAAAACGTCGATGAG-3′), *CTNNB1* (forward: 5′-GCTTTCAGTTGAGCTGACCA-3′; reverse; 5′-CAAGTCCAAGATCAGCAGTCTC-3′), and housekeeping gene *TBP* (forward: 5′-GAGCTGTGATGTGAAGTTTCC-3′; reverse: 5′-TCTGGGTTTGATCATTCTGTAG-3′). The QuantStudio 3 system was used to determine the amount of target mRNA in each sample, estimated using the 2^−ΔΔCt^ relative quantification method. Gene expression levels were normalized against human *TBP* gene levels in each sample.

### 4.6. Statistical Analysis

Statistical tests were performed using GraphPad Prism 8. Results are shown as the mean ± SD of data from three independent samples (*n* = 3) in each study group. The statistical significance of data was determined using one-way ANOVA (multiple comparisons by Dunnett’s test) and, in all cases, a *p*-value < 0.05 was considered to be statistically significant (* *p* < 0.05, ** *p* < 0.01, *** *p* < 0.001, **** *p* < 0.0001).

## Data Availability

The data presented in this study are available by request.

## Supplementary Materials

The supporting information can be downloaded at https://www.mdpi.com/article/10.3390/ijms241914635/s1.
