# Supplementary material for: Low Levels of Amyloid Precursor Protein (APP) Promote Neurogenesis and Decrease Gliogenesis in Human Neural Stem Cells
Source: Int J Mol Sci. 2023 Sep 27;24(19):14635. doi: 10.3390/ijms241914635 (PMC10572469; doi:10.3390/ijms241914635)
Supplement: Supplementary file 1 [file ijms-24-14635-s001.zip › ijms-2590639-supplementary.pdf]

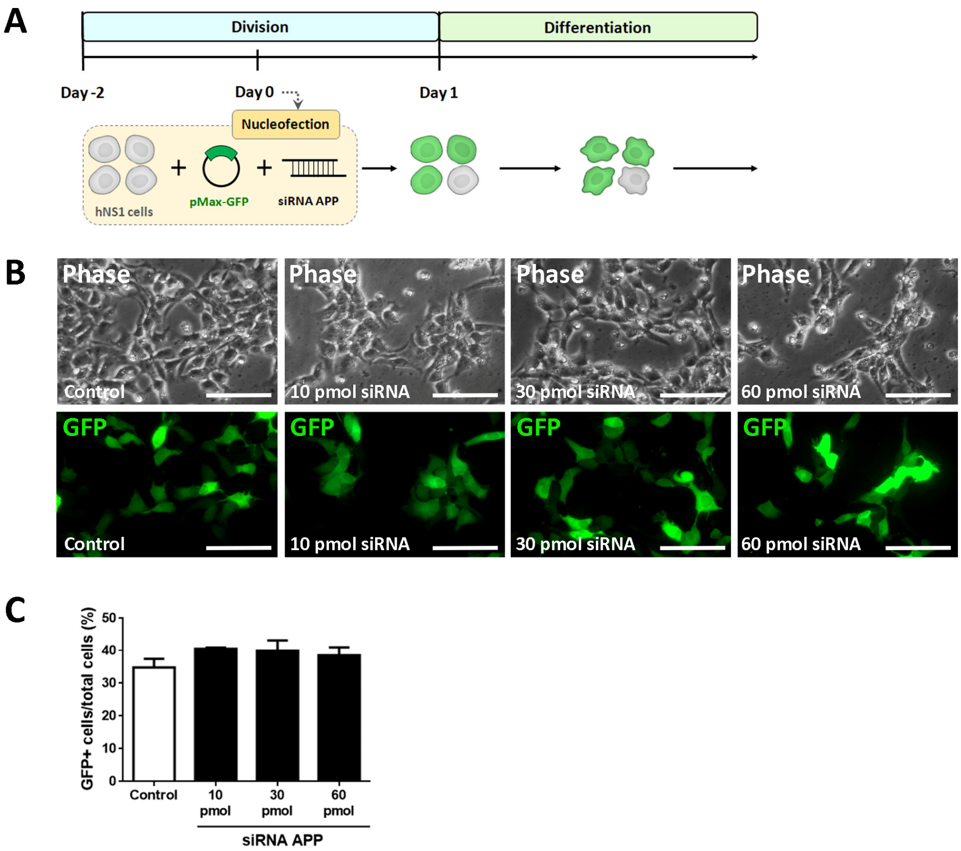

Supplementary Figure S1: Nucleofection efficiency in hNS1 cells. (A) Schematic representation of co-nucleofection experiment (siRNA APP and pMax-GFP) done in hNS1 cells. (B) Representative images in phase contrast and the corresponding field with GFP+ cells in control hNS1 cells and hNS1 cells with APP silencing (10 pmol, 30 pmol, 60 pmol siRNA). Scale bar = 50µm. (C) Percentage of GFP+ cells with respect to total cells (phase contrast) in all study groups (control, 10 pmol, 30 pmol, 60 pmol siRNA). Data represent mean ± SD (n=3 for three independent samples). Statistical analysis was performed using one-way ANOVA (multiple comparison of each group vs control group).
